# *Helicobacter pylori* Subdues Cytokine Signaling to Alter Mucosal Inflammation *via* Hypermethylation of Suppressor of Cytokine Signaling 1 Gene During Gastric Carcinogenesis

**DOI:** 10.3389/fonc.2020.604747

**Published:** 2021-01-25

**Authors:** Iqra Jan, Rafiq A. Rather, Ifra Mushtaq, Ajaz A. Malik, Syed Besina, Abdul Basit Baba, Muzamil Farooq, Tahira Yousuf, Bilal Rah, Dil Afroze

**Affiliations:** ^1^Department of Immunology and Molecular Medicine, Sher-I-Kashmir Institute of Medical Sciences, Srinagar, India; ^2^Department of Advanced Centre for Human Genetics, Sher-I-Kashmir Institute of Medical Sciences, Srinagar, India; ^3^Department of General and Minimal Invasive Surgery, Sher-I-Kashmir Institute of Medical Sciences, Srinagar, India; ^4^Department of Pathology, Sher-I-Kashmir Institute of Medical Sciences, Srinagar, India

**Keywords:** mucosal inflammation, *H. pylori*, *SOCS1*, hypermethylation, carcinogenesis

## Abstract

*Helicobacter pylori* infection has been associated with the onset of gastric mucosal inflammation and is known to perturb the balance between T-regulatory (Treg) and T-helper 17 (Th17) cells which causes a spurt of interleukin 17 (IL17) and transforming growth factor-β (TGF-β) from Th17 and Treg cells within the gastric milieu. IL17 instigates a surge of interleukin 6 (IL6) from T-helper 1 (Th1) and T-helper 2 (Th2) cells. Further, *H. pylori* infection is known to stimulate the atypical DNA methylation in gastric mucosa. However, the precise role of cytokine signaling in induction of epigenetic modifications during gastric carcinogenesis is vaguely understood. In this study, patient samples from were examined using real-time polymerase chain reaction (qPCR), PCR, methylation-specific (MS)-PCR, and enzyme-linked immunosorbent assays. We found that *H. pylori* infection augments the production of interleukin 10 (IL10), IL6, and TGF-β in the gastric milieu and systemic circulation. Together with the IL6/IL10 mediated hyperactivation of the JAK/STAT pathway, *H. pylori* infection causes the inactivation of suppressor of cytokine signaling 1 (*SOCS1*) gene through the hypermethylation of the promoter region. This study signifies that *H. pylori*-mediated epigenetic silencing of *SOCS1* in concert with inflammatory cytokines miffs hyperactivation of the JAK/STAT cascade during gastric carcinogenesis.

## Introduction

Gastric cancer arises as a consequence of series of genetic, epigenetic and metabolic alteration in the mucus-producing cells on the inner surface of the gastric mucosa. Gastric cancer cells express a plethora of growth factor and cytokine receptor systems that form a highly complex interaction network between cancer cells and stromal cells within tumor microenvironment and impart therapeutically-resistant phenotype to gastric tumors. Further, a very small population of cancer-stem cells (CSCs) exists within gastric tumor microenvironment which play fundamental role in tumor initiation, progression, metastasis and recurrence. Recently, mucosal gastric microbiota has received tremendous attention due to its effect on the growth of gastric cancer and cancer-associated signaling cascades (1). Because of the acidic pH within stomach, gastric mucosa is typically considered as an unfriendly environment for microbial growth. Nevertheless, gram-negative bacterium *Helicobacter pylori* successfully reside in the gastric mucosa and damages gastric epithelial cells (2). *H. pylori* protect itself from acidic pH by epithelial secretions of bicarbonate buffer system and urea and the ammonia created by urease action and by tight attachment to mucosal glycan receptors (3). Although short-term colonization of *H. pylori* in gastric mucosa is asymptomatic (4), approximately 10% of *H. pylori*-infected individuals develop peptic ulcers, 1 to 3% develop gastric adenocarcinoma and <0.1% develop mucosa associated lymphoid tissue (MALT) lymphoma (5). However, persistent colonization of *H. pylori* in gastric mucosa significantly enhances the probability of developing gastric ulcers, MALT lymphoma and gastric carcinoma (6). Notably, *H. pylori* infection mediated precancerous lesions are immunopathological in origin (7).

In response to *H. pylori* infection, a group of cytokines such as IL17, IL6 and transforming growth factor-β (TFG-β) are released from immune-sensitive cells like T-helper 1 (Th1), T-helper 2 (Th2), T-helper 17 (Th17) and T-regulatory (Treg) cells within gastric milieu. It has been recognized that IL17 produced by Th17 cells causes activation of Th1 and Th2 cells and subsequently causes release of IL6 from these cells in gastric milieu (8, 9). IL6 and TGF-β are considered as central mediators in *H. pylori*-associated pathogenesis and are capable of directly influencing JAK/STAT signaling cascade (10). Among the key genes that are involved in *H. pylori* pathogenicity, phosphoglucosamine mutase gene (*glmM*) and cytotoxin associated gene A (*CagA*) virulence genes are strong predictors of clinical outcomes (11). The interaction of virulence factors, host genetic factors and environmental factors contribute to *H. pylori*-associated pathogenesis. Although gastritis is induced by all strains of *H. pylori*, not all *H. pylori* strains are capable of causing the gastric carcinoma. Only the *H. pylori* strains containing *Cag* PAI (*CagA^+^*) increases the probability of developing gastric cancer (12–14). *H. pylori*-carrying *CagA* gene cause genetic instability in gastric mucosa cells, usually modulating activities of different genes (15).

The immune response to the *H. pylori* infection in the gastric mucosa is typically associated with the release of inflammatory and anti-inflammatory cytokines from different types of immune cells like Th1, Th2, Th17, macrophages, monocytes, mast cells and neutrophils. Many of these cytokines such as IL1, IL2, IL6, IL10, IL17, IL23, TNF-α, TGF-β, IFN-γ, CXCL12, and CXCL4 are secreted both at the site of infection and in the general blood circulation (16). Specific membrane receptors are expressed by gastric epithelial cells that help to transmit the signal carried by different cytokines to the cell interior (17). Cytokines act on broad range of cell types wherein they bind to their cognate membrane receptors and modulate downstream effectors in the signalling cascade (18). Many cytokines activate janus kinase 2 (JAK2) which in turn phosphorylates signal transducers and activators of transcription 3 (STAT3). Phosphorylated STAT3 enter the nucleus and bind specific regulatory sequences to activate or repress transcription of target genes. Besides acting as a transcription factor, STAT3 has recently been shown to act as a marker for gastric carcinoma progression (19). An important negative regulator of JAK/STAT signaling cascade is suppressor of cytokine signaling-1 (SOCS1) which interacts with phosphotyrosine residues on JAK kinases to interfere with the activation of STAT proteins or other signaling intermediates. Interestingly, inactivation of the SOCS1 has been proposed to be responsible for oncogenesis in gastric mucosa (20). IL6 and TFG-β are vital for regulating SOCS1 expression. It has been reported that hypermethylation of SOCS1 significantly retarded growth and proliferation of variety of gastric cancer cell lines (21). The present study has brought out that *H. pylori*-mediated epigenetic silencing of *SOCS1* in concert with inflammatory cytokines causes hyperactivation of the JAK/STAT cascade during gastric cancer development.

## Materials and Methods

### Patient Sample Collection

In this study we enrolled fifty two gastric cancer patients whose disease status was confirmed by clinical and histopathological examination from the Department of General and Minimal Invasive Surgery, SKIMS, Srinagar, Kashmir. Sample collection was completed in accordance with norms and regulations of Institutional Ethics Committee (IEC-SKIMS). Ethical clearance for the use of human samples was obtained in accordance with the declaration of Helsinki. Only the gastric cancer patients with upfront gastrectomies were included in the study. All the enrolled patients gave informed consent for collecting the samples. The number of patient samples was calculated by open-Epi statistical software. The power of study was maintained at >80% and confidence interval was maintained at 95%.

### DNA Extraction

The extraction of whole genomic DNA from gastric cancer and adjacent normal tissues was achieved by using phenol/chloroform-isoamyl (PCI) method. Briefly, tissue samples were homogenized and solubilized in DNA lysis buffer [Tris-HCl (10 mM, pH 8.0), NaCl (100 mM), EDTA (5 mM), triton X-100 (5%), SDS (0.25%), RNase A (200 μg/mL)] and incubated at 37°C overnight. After incubation, tissue lysate was treated with proteinase K (200 μg/mL) for one and a half hour. Subsequently, DNA was extracted with phenol/chloroform-isoamyl alcohol (25:24:1). The concentration of DNA was measured by Nano Drop 2000. A_260_/A_280_ ratio was used to ascertain the purity of DNA. The purity and quality of DNA was further determined by 2% agarose gel electrophoresis. After the extraction process, specific primers for *glmM* and *CagA* bacterial genes were used to determine the infection status of gastric cancer.

### RNA Extraction

TRIzol reagent (Invitrogen) was used to extract total RNA from gastric cancer and adjacent normal tissues. The concentration of the total RNA used for the synthesis of cDNA was >1µg. The concentration of total RNA was determined by Nano Drop 2000. MMLV reverse transcriptase kit (Invitrogen) was used for the reverse transcription of RNA into cDNA. Next, we analyzed the real time quantitative expression of IL6, IL10, JAK2, STAT3, and β-actin using designed exon specific primers of each gene. β-actin was used as an internal control. In order to assess the purity and quality of RNA, small samples of RNA were electrophoresed on 2% agarose gel, stained with ethidium bromide and visualized under ultraviolet illumination. The quality of RT-PCR products was assessed using the same technique.

### ELISA

A solid phase immunoassay kit from “BOSTER Antibody & ELISA Experts” were used to measure serum cytokine level. Briefly, to measure cytokine levels in serum, standard and test samples were added to 96-well plate followed by the addition of biotinylated detection antibody. After this, wells were washed with tris-buffered saline (TBS) buffer and avidin–biotin–peroxidase complex (ABC-HRP) was added. Any unbound ABC-HRP can be washed off by TBS. Subsequently, 3,3’,5,5’-tetramethylbenzidine (TMB) substrate was added which is metabolized to a blue colored product by HRP enzyme. The reaction is stopped by the addition of acidic stop solution, resulting in change of color from blue to yellow. The optical density of yellow color is linearly proportional to the amount of cytokine present in the sample which can be measured at 450 nm using a plate reader. The biomarkers evaluated from the gastric cancer patient serum were IL6, IL10, TNF-α, and TGF-β.

### Methylation-Specific (MS)-PCR for *SOCS-1* and *STAT-3* Gene Promoter Hypermethylation

MS-PCR analysis of *SOCS-1* and *STAT-3* gene promoters was carried out using methylation-specific primer sets. About 2–3 µl of the bisulphite converted DNA from each sample (including both gastric cancer and adjacent normal tissue) was used for the setting up of the MS-PCR. Universal methylated human DNA (Zymo Research, USA) was used as positive control for methylated alleles whereas DNA from normal healthy subjects was used as a control for unmethylated alleles. Water was used as a negative PCR control in both reactions.

## Statistical Analysis

Statistical analysis of numerical data was performed by using non-parametrical statistical analysis tools including Mann-Whitney U test and Kruskal-Wallis tests. Overall survival analysis was carried out by using Kaplan-Meir analysis. Software used was SPSS ver. 23.

## Results

### Prevalence and General Characteristics of *H. pylori*-Infected Gastric Cancer Patients

In this study, a total of 52 histopathologically confirmed gastric cancer specimens and 52 adjacent noncancer tissues were obtained from General and Minimal Invasive Surgery, SKIMS, Srinagar, Kashmir, India to understand the mechanism of gastric carcinogenesis in ethnic Kashmiri population. First, we found that clinical characteristics such as age (> 50 years), gender, family history, active life style, and rural dwelling were significantly associated with the occurrence of gastric cancer (**Table 1**). Kashmiri population is often exposed to pesticides. Therefore, to exclude the involvement of pesticides in the development of gastric carcinoma, pesticide exposure was taken into consideration. However, pesticide exposure did not reveal any association with the development of gastric carcinoma. Our study revealed that most of the patients were above 50 years of age and male individuals with smoking habit appeared increasingly susceptible to the gastric carcinoma.

**Table 1:** Characteristics of H. pylori-infected gastric cancer patients.

Patient characteristics	Cases n=52	p Value
Age		
≤50	7 (13.5%)	
>50	45 (86.5%)	0.0001
Gender		
Male	34 (65.4%)	
Female	18 (34.6%)	0.06
Family History		
With History	36(69.2%)	
Without History	16(30.8%)	0.02
Smoker		
Yes	33 (63.5%)	
No	19 (36.5%)	0.1
Life Style		
Active	49 (94.2%)	
Sedentary	3 (5.8%)	0.0001
Body Mass		
Normal	41 (78.8%)	
Obese	3 (5.8%)	
Lean	8 (15.4%)	0.08
Socioeconomic Status		
High	4 (1.9%)	
Low	18 (34.6%)	
Middle	33 (63.5%)	0.3
Residence		
Rural	42(80.8%)	
Urban	10(19.2%)	0.0002
Pesticide Exposure		
Yes	23(44.2%)	
No	29(55.8%)	0.4

### Clinical Complaints of Gastric Cancer Patients

Data from gastric cancer patients (n=52) was examined, and it was observed that abdominal pain was the most noticeable clinical complaint experienced by majority of gastric cancer patients. In fact, abdominal pain was the single most noted pain and therefore should not be ignored while examining a patient. However, the patients also revealed one or more of the clinical complaints such as vomiting, anorexia, generalized weakness, weight loss, dysphagia, dyspepsia, malena and upper gastrointestinal (UGI) bleed. The clinical complaints have been arranged in the ascending order of their occurrence among the enrolled patients. These clinical complaints are not mutually exclusive, but many of these may develop in a single individual patient (**Supplementary Figure 1**).

### CagA and glmM Status of Gastric Tumor Tissues Indicated Existence of a Strong Connection Between *H. pylori* Infection and Gastric Carcinoma

*H. pylori* affect the gastric epithelium of nearly 50% of world’s human population. Although a strong correlation exists between the *H. pylori* infection and gastric carcinoma, the exact mechanism of *H. pylori*-induced gastric carcinoma has not been precisely determined. CagA is an oncoprotein that plays an important role in gastric carcinogenesis whereas glmM is often used an indicative of the bacterial load. PCR analysis of CagA and glmM in gastric cancer tissues indicated that majority of gastric tumors are positive for CagA (~92%) and glmM (~67%) (**Supplementary Figures 2** and **3**). This discrepancy may be due to more prominent role of CagA in atropic gastritis which leads to increased risk of gastric carcinoma than glmM which is the responsible for chronic active gastritis only. Our results suggest that CagA oncoprotein (an indicative of bacterial virulence) plays a greater role in gastric carcinogenesis than glmM (an indicative of the bacterial load).

### Inflammation Induced by *H. pylori* in Gastric Epithelium Triggers Gastric Carcinogenesis

**Figure 1:**
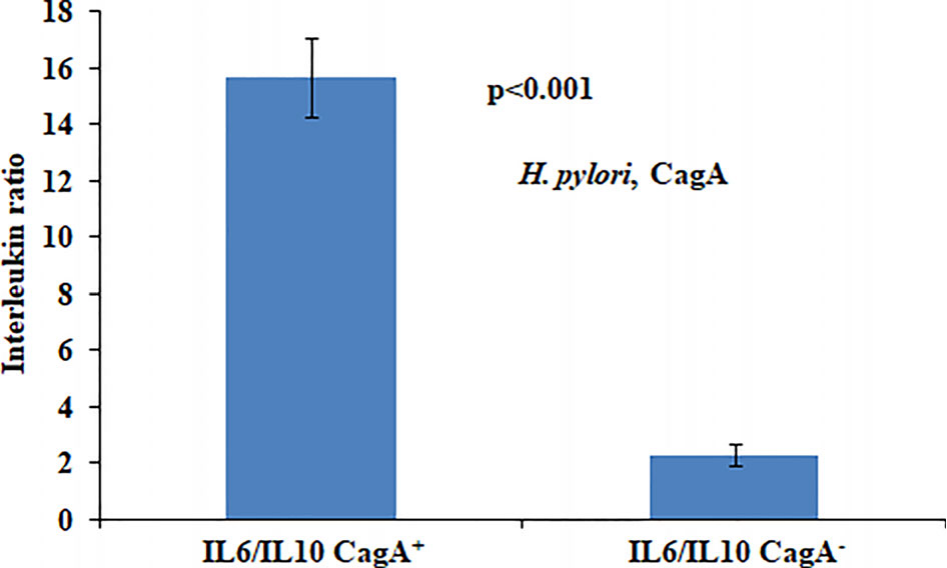
Ratio of circulatory cytokines (IL6/IL10) vis-à-vis local *H. pylori* infection status.

In the present study we found that presence of CagA was significantly associated with the aberrantly high ratio of IL6 and IL10. High IL6/IL10 ratio in *H. pylori*-infected patients indicated that pro-inflammatory cytokines play a fundamental role in gastric cancer development (**Figure 1**).

### Methylation of SOCS-1 in Gastric Cancer Tissues and Adjacent Normal Tissues

As discussed above, we found that that IL6 was substantially expressed compared to IL10 in *H. pylori*-infected tumor tissues. Since IL6 upregulates *SOCS-1*, which is a negative regulator of JAK-STAT signaling cascade, we next examined the methylation pattern of SOCS-1 gene promoter in tumor tissues. It is interesting to note that DNA methylation occurs when the cytosine in cytosine-guanine dinucleotides is converted into methyl-cytosine. Binding of transcription factors to such gene promoter regions is blocked due to their hypermethylation, thus rendering gene silencing or loss of expression. We found that *SOCS-1* gene promoter was methylated in majority of tumor specimens (partially methylated in ~75% and fully methylated in ~15% of tumor specimens) (**Supplementary Figure 4**). *SOCS-1* hypermethylation results in overactivation of JAK/STAT signaling cascade which in turn results abnormal proliferation of gastric mucosal cells. It is worthy to mention here that hypermethylation was also found in adjacent normal tissues suggesting that methylation may be involved during the early phases of gastric carcinogenesis as well.

### Correlation of Clinical Characteristics of Gastric Cancer Patients With SOCS1 Hypermethylation and Mediators of the JAK/STAT Pathway

On correlating patient characteristics with virulence marker of *H. pylori* infection (CagA and glmM), we found that male individuals were increasingly prone to *H. pylori* infection. Further, we analyzed that the patients admitted with clinical symptoms of generalized weakness, weight loss and malena were more susceptible to *H. pylori*-induced development of gastric cancer (**Table 2**). Furthermore, we found that *H. pylori*-infected patients acquired intestinal type gastric cancer and entered into metastatic stage III of cancer progression. In this study, we worked out that *H. pylori* infection leads to higher mRNA expression of IL-6, JAK-2 and STAT-3 at the local site of infection which better explains the progression of gastric cancer at the molecular level. We found that most of the patients (~86.5%) with *H. pylori*-infected gastric cancer tissues had hypermethylation of promoter region of SOCS-1 gene which could be a possible reason for the devastating nature of gastric cancer (**Table 3**). Besides the higher levels inflammatory cytokines at the local area of infection by *H. pylori*, we also evaluated that concentration of inflammatory cytokines like IL-6 and TGF-β was raised in the general circulation predicting the aggressive nature of gastric cancer.

**Table 2:** Correlation of *H. pylori* infection with general characteristics/clinical complaints/histopathological characteristics of patients.

	*H. pylori* infection (mean rank)		p value for *H. pylori* (+ve) versus *H. pylori* (-ve)
	+ve	-ve	
Clinical Parameters of patients			
Gender	34.83	25.41	0.05
Clinical complaints of patients			
General weakness	27.83	10.50	0.009
Weight loss	27.38	16.00	0.05
Malena	31.00	24.31	0.02
Histopathological characteristics			
Intestinal type GC (Lauren’s classification)			0.05
HPE (Metastatic tumor)	27.89	15.83	0.04
Stage III	41.38	25.26	0.007

**Table 3:** Correlation of *H. pylori* infection with mRNA expression, methylation profile and serum cytokine levels of gastric cancer patients.

	*H. pylori* infection (mean rank)		p value for *H. pylori* (+ve) versus *H. pylori* (-ve)
	+ve	-ve	
mRNA expression in the local tumor region			
IL-6	31.79	23.41	0.05
JAK-2	31.97	23.33	0.05
STAT-3	32.79	24.41	0.05
Methylation status			
SOCS-1	28.73	17.08	0.05
Serum cytokine levels			
IL-6	16.48	6.67	0.05
TGF-β	21.64	13.63	0.03
IL10	24.91	27.76	0.2

### Association of *H. pylori* Status With Survival

**Figure 2:**
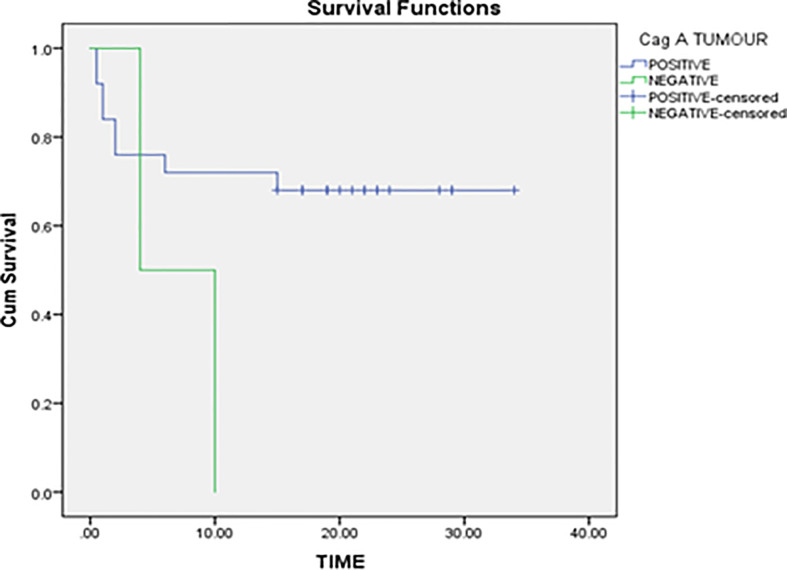
Correlation of *H. pylori* infection with overall survival of gastric cancer patients.

In the non-parametric analysis, absence of *H. pylori* infection was associated with better gastric cancer-specific survival. Our data revealed that the mean survival rank of *H. pylori* negative patients was ~22 while the mean survival rank of *H. pylori* positive patients was ~12 (**Figure 2**). Kaplan–Meier survival curves indicated that *H. pylori* infection can be a potential prognostic biomarker for gastric cancer.

## Discussion

Gastric cancer is an intricate disease which develops as a consequence of complex interaction between environmental, habitual, dietary, genetic, and epigenetic factors. A proper analysis of different risk factors associated with gastric cancer development has helped a lot in understanding the possible mechanism of gastric carcinogenesis. A recently conducted meta-analysis revealed that *H. pylori* infection is the central reason for the growth of gastric cancer and affects males more aggressively than females (22).

*H. pylori* infection has been shown to trigger multiple motor neuron disorders which cause generalized weakness experienced by the *H. pylori*-infected gastric cancer patients (23). Apart from generalized weakness, the most common clinical complaints experienced by the *H. pylori*-infected gastric cancer patients, included dyspepsia, dysphagia, vomiting, malena, abdominal pain and weight loss. These findings are in agreement with our results. *H. pylori*-induced chronic active gastritis has been associated with the initiation of intestinal type of gastric adenocarcinoma. In our study, we found that gastritis-cum-abdominal pain was the most common complaint experienced by gastric cancer patients. Thus, functional abdominal pain of *H. pylori*-infected patients should not be ignored. In addition, we found that gastric cancers, that we studied, were majorly of intestinal-type and originated in the antropyloric region of the stomach. Further, it has been reported that *H. pylori*-infected gastric cancer patients usually reach to stage III of the disease (24). Using mouse models of gastric cancer, it has been reported that upregulation of IL-6 and activation of STAT-3 in *H. pylori*-infected gastric cancer ensues to activation of JAK/STAT signaling cascade. Several studies have been conducted to understand the underlying mechanism of JAK/STAT activation (25). Recently, it has been found that hyperactivation of JAK/STAT in *H. pylori*-infected gastric cancer patients occur *via* hypermethylation of promoter region of SOCS1 gene (21). This study is strongly in agreement with our finding. We evaluated that chronic *H. pylori* infection leads to the release of numerous inflammatory mediators including chemokines [IL8, macrophage chemotactic protein (MCP)-1, growth-regulated oncogene (GRO)-α] and cytokines [IL1-β, tumor necrosis factor (TNF)-α, IL6, IL12, interferon (IFN)-γ], which enter into the general blood circulation and exert a systemic effect (23).

In summary, hypermethylation of the promoter region of the *SOCS-1* gene together with high production of endogenous IL6 and TGF-β leads to hyperactivation of JAK/STAT in gastric tumors. The increase in STAT3 activity concomitant with overexpression of IL6 and TGF-β suggests that aberrant activation of JAK-STAT signaling cascade is a key event for gastric carcinogenesis. Therefore, the release of inflammatory as well as regulatory cytokines by the *H. pylori*-infected mucosal immune cells at the local area as well as in the general circulation can be considered as an important trigger for the initiation and progression of gastric cancer. The present study has brought out that *H. pylori*-mediated cytokine signaling promotes gastric cancer development through hypermethylation of *SOCS-1* gene promoter and aberrant activation of JAK-STAT cascade. Therefore, concurrent eradication of *H. pylori* and inhibition of JAK-STAT signaling cascade by applying specific JAK2 inhibitors may unravel new therapeutic strategies against the gastric cancer in ethnic Kashmiri population. This study provides us with ample clues regarding the different steps and factors which we can tackle therapeutically in order to stop this deadly disease from progression.

## Data Availability Statement

The raw data supporting the conclusions of this article will be made available by the authors, without undue reservation.

## Ethics Statement

The studies involving human participants were reviewed and approved by Institutional Ethics Committee (IEC-SKIMS). The patients/participants provided their written informed consent to participate in this study.

## Author Contributions

IJ performed the experiments with the assistance from DA, RR, IM, AM, SB, BB, MF, TY, and BR. IJ, DA, and RR analyzed the data. DA acquired the funding and supervised the project. All authors contributed to the article and approved the submitted version.

## Funding

This research was funded by the Science and Engineering Research Board (SERB), Department of Science and Technology, New Delhi, India (Grant Number: EMR/2016/004794) and intramural grants from SKIMS, Soura, Srinagar (India).

## Conflict of Interest

The authors declare that the research was conducted in the absence of any commercial or financial relationships that could be construed as a potential conflict of interest.

## Supplementary Material

Supplementary Figure 1 Clinical complications of gastric cancer patients

Supplementary Figure 2 PCR products of CagA gene in gastric cancer tissues along with their adjacent normal tissues run on 2% agarose gel.

Supplementary Figure 3 PCR amplified products of *GlmM* gene in gastric cancer tissues along with their adjacent normal tissues run on 2% agarose gel.

Supplementary Figure 4 MS-PCR products of SOCS1 in normal (methylated, unmethylated) and gastric cancer tissues (methylated & unmethylated). **(A)** Lane1-M(N), Lane2-UM(N), Lane3-M(N), Lane4-UM(N), Lane5-M(N), Lane6-UM(N), Lane7-M(N), Lane8-UM(N), Lane9-M(N), Lane10-UM(N), Lane11-M(N), Lane12-UM(N), Lane13-50bp ladder. **(B)** Lane1-M (N), Lane2-UM(N), Lane3-M(N), Lane4-UM(N), Lane5-M(N), Lane6-UM(N), Lane7-M(N), Lane8-UM (N), Lane9-M(N), Lane10-UM(N), Lane11-M(N), Lane12-UM(N), Lane13-50bp ladder. **(C)** Lane1-M(N), Lane2-UM(N), Lane3-M(N), Lane4-UM(N), Lane5-M(N), Lane6-UM(N), Lane7-M(N), Lane8-UM(N), Lane9-M(N), Lane10-UM(N), Lane11-M(N), Lane12-UM(N), Lane13-50bp ladde.
