# Supplementary figures and images for: Helicobacter pylori Subdues Cytokine Signaling to Alter Mucosal Inflammation via Hypermethylation of Suppressor of Cytokine Signaling 1 Gene During Gastric Carcinogenesis
Source: Front Oncol. 2021 Jan 25;10:604747. doi: 10.3389/fonc.2020.604747 (PMC7868987; doi:10.3389/fonc.2020.604747)

Supplementary Figure 1

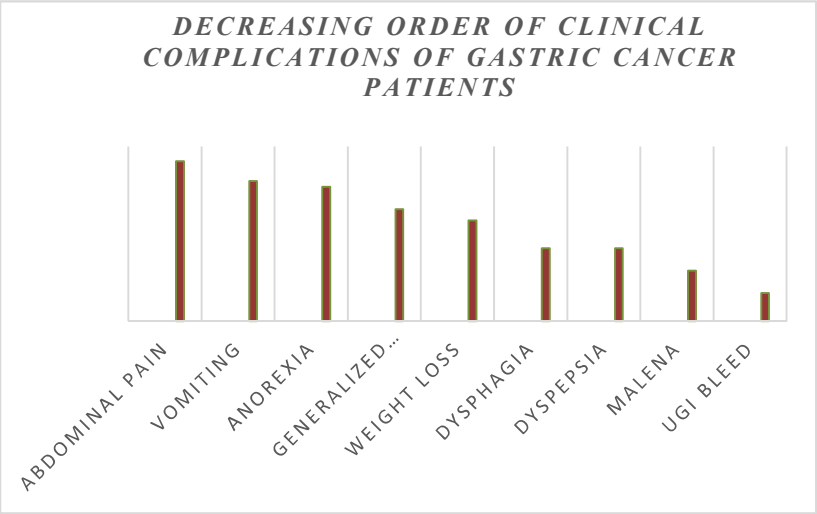

Supplementary Figure 2

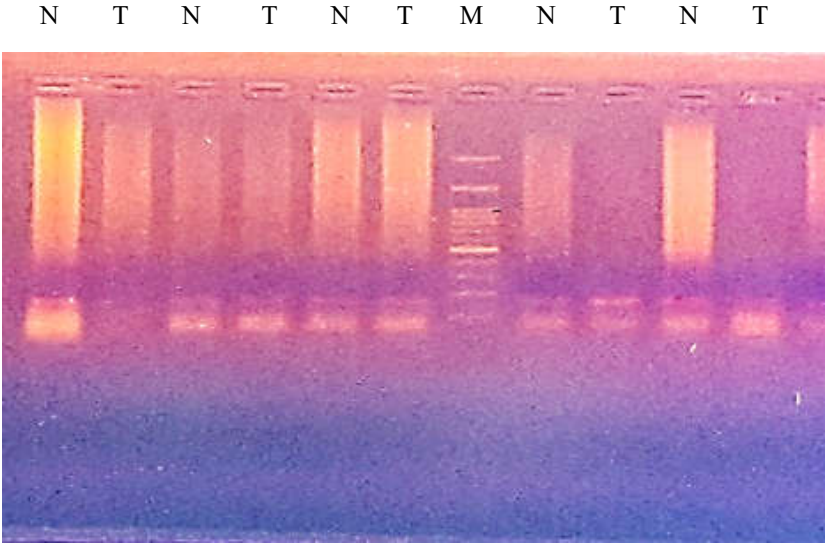

Supplementary Figure 3

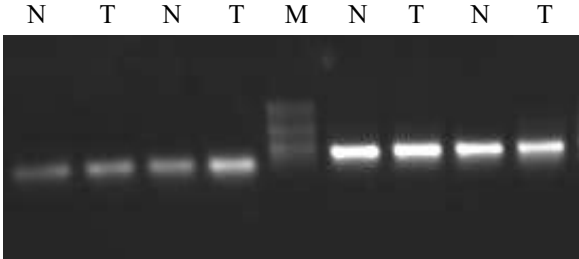

### Supplementary Figure 4

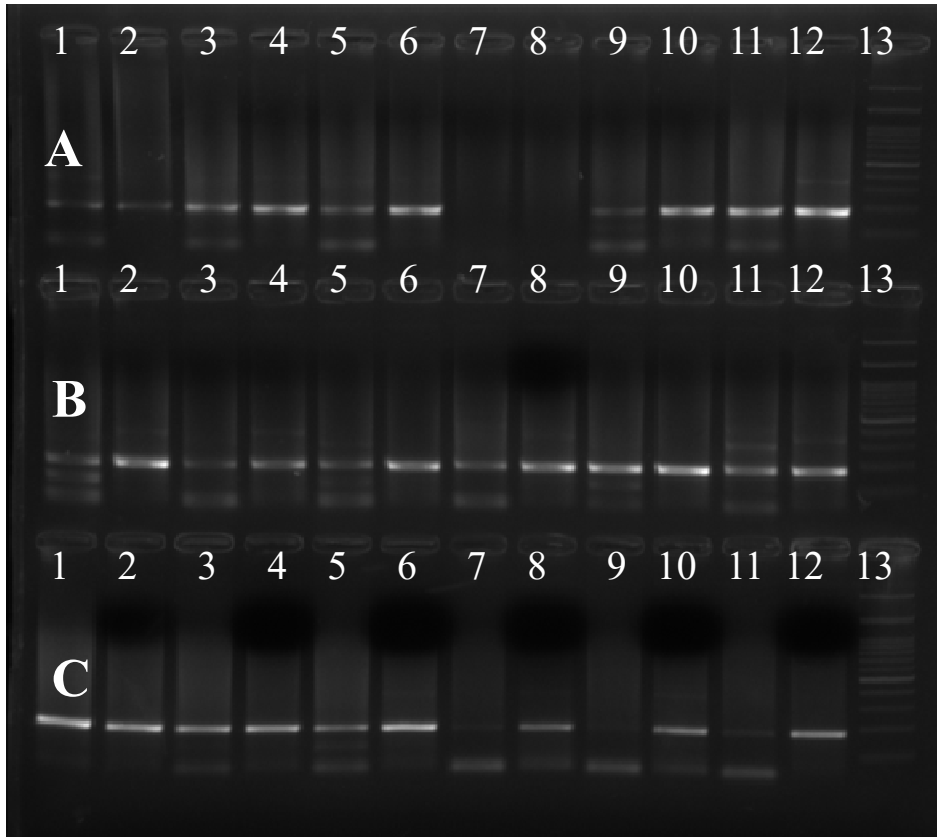

Supplement: Supplementary Figure 1 — Clinical complications of gastric cancer patients [file Image_1.pdf]
