# Supplementary material for: Helicobacter pylori Subdues Cytokine Signaling to Alter Mucosal Inflammation via Hypermethylation of Suppressor of Cytokine Signaling 1 Gene During Gastric Carcinogenesis
Source: Front Oncol. 2021 Jan 25;10:604747. doi: 10.3389/fonc.2020.604747 (PMC7868987; doi:10.3389/fonc.2020.604747)
Supplement: Supplementary file 2 [file DataSheet_1.pdf]

***Helicobaacter pylori* subdues cytokine signaling to alter mucosal inflammation via hypermethylation of suppressor of cytokine signaling 1 (*SOCS1)* gene during gastric carcinogenesis**

Iqra Jan, Rather A. Rafiq, Ifra Mushtaq, Ajaz A. Malik, Syed Besina, Abdul Basit Baba, Muzamil Farooq, Tahira Yousuf, Bilal A Rah, Dil-Afroze

Supplementary Figure Legends

Supplementary Figure 1. Clinical complications of gastric cancer patients

Supplementary Figure 2. PCR products of CagA gene in gastric cancer tissues along with their adjacent normal tissues run on 2% agarose gel.

Supplementary Figure 3. PCR amplified products of *GlmM* gene in gastric cancer tissues along with their adjacent normal tissues run on 2% agarose gel.

Supplementary Figure 4. MS-PCR products of SOCS1 in normal (methylated, unmethylated) and gastric cancer tissues (methylated & unmethylated)

A) Lane1-M(N), Lane2-UM(N), Lane3-M(N), Lane4-UM(N), Lane5-M(N), Lane6-UM(N), Lane7-M(N), Lane8-UM(N), Lane9-M(N), Lane10-UM(N), Lane11-M(N), Lane12-UM(N), Lane13-50bp ladder

B) Lane1-M (N), Lane2-UM(N), Lane3-M(N), Lane4-UM(N), Lane5-M(N), Lane6-UM(N), Lane7-M(N), Lane8-UM (N), Lane9-M(N), Lane10-UM(N), Lane11-M(N), Lane12-UM(N), Lane13-50bp ladder

C) Lane1-M(N), Lane2-UM(N), Lane3-M(N), Lane4-UM(N), Lane5-M(N), Lane6-UM(N), Lane7-M(N), Lane8-UM(N), Lane9-M(N), Lane10-UM(N), Lane11-M(N), Lane12-UM(N), Lane13-50bp ladder
